# Applicability of Bolton’s Analysis and Regression-Based Tooth-Size Prediction Methods in a Young Adult Cohort

**DOI:** 10.7759/cureus.110336

**Published:** 2026-06-06

**Authors:** Beenamol Boben, Ravindra Vangala, Divya Swapna Maraka, Pavalla Venkata Sai Krishna, Uday T Kumar, Vijayalakshmi Besta, Prasad Mandava, Gowri Sankar Singaraju

**Affiliations:** 1 Dentistry, Narayana Dental College and Hospital, Nellore, IND; 2 Orthodontics and Dentofacial Orthopaedics, Gandhi Institute of Technology and Management (GITAM) Dental College and Hospital, Visakhapatnam, IND; 3 Orthodontics and Dentofacial Orthopaedics, Narayana Dental College and Hospital, Nellore, IND

**Keywords:** bolton analysis, incisor prediction, interarch relationship, mesiodistal width, orthodontics, regression analysis, tooth-size discrepancy

## Abstract

Introduction

Accurate assessment of interarch tooth-size relationships is essential for orthodontic diagnosis, space analysis, and treatment planning. Bolton’s analysis and regression-based methods such as Tonn’s and Abhi’s formulas are commonly used to predict incisor dimensions, but their applicability across different populations remains uncertain.

Methods

This observational validation study was conducted on 56 dental study models of young adults aged 18-25 years. Mesiodistal widths of maxillary and mandibular anterior teeth were measured using a digital Vernier caliper. Actual tooth dimensions were compared with values predicted using Bolton’s anterior ratio, Tonn’s formula, and Abhi’s formula. Statistical analysis included paired t-test, Pearson correlation analysis, Bland-Altman agreement analysis, and linear regression analysis.

Results

The mean anterior Bolton’s ratio was 78.02% ± 2.05%, significantly higher than Bolton’s standard value of 77.2% (p = 0.003). Abhi’s formula underestimated mandibular incisor width by 0.67 mm (p < 0.001), whereas Tonn’s formula overestimated maxillary incisor width by 1.67 mm (p < 0.001). Bland-Altman analysis showed a mean bias of +1.67 mm for Tonn’s formula, with 95% limits of agreement from −0.30 to +3.70 mm, and a mean bias of −0.67 mm for Abhi’s formula, with 95% limits of agreement from −2.40 to +1.10 mm. Linear regression analysis showed a moderate positive relationship between the sum of maxillary and mandibular incisor widths (r = 0.725, R² = 0.526, standard error of estimate (SEE) = 0.92 mm). The derived equation showed good internal validation, with the predicted anterior Bolton’s ratio of 78.11% showing no significant difference from the actual anterior Bolton’s ratio of 78.02% (p = 0.977).

Conclusions

Bolton’s analysis, Tonn’s formula, and Abhi’s formula demonstrated significant bias in the present cohort. Population-specific regression equations showed improved predictive accuracy and may provide more reliable guidance for orthodontic diagnosis and treatment planning.

## Introduction

Mesiodistal tooth width plays an important role in achieving ideal occlusion, functional harmony, and esthetic balance during orthodontic treatment. Proper coordination between maxillary and mandibular tooth dimensions is essential for maintaining ideal overjet, overbite, intercuspation, and long-term stability. Bolton introduced anterior and overall tooth-size ratios to evaluate interarch tooth-size discrepancies, and these ratios continue to remain one of the most widely used diagnostic tools in orthodontics [1]. Even small discrepancies in anterior tooth dimensions may result in crowding, spacing, midline deviations, compromised intercuspation, and poor smile esthetics [1,2]. Therefore, accurate estimation of tooth dimensions is important during orthodontic diagnosis, space analysis, and restorative treatment planning, particularly when direct measurement of the tooth is not possible [3].

The importance of tooth-size prediction becomes more significant in patients presenting with developmental anomalies affecting the anterior dentition, especially peg-shaped lateral incisors and congenitally missing incisors [4]. Peg-shaped maxillary lateral incisors represent a localized form of microdontia associated with reduced mesiodistal width and altered crown morphology. A meta-analysis involving more than 87,000 individuals reported a global prevalence of peg-shaped lateral incisors of approximately 1.8%, with a relatively higher prevalence among Asian populations [5]. Congenital absence of maxillary lateral incisors is also among the most frequently encountered developmental dental anomalies in orthodontic practice [4]. Indian studies have similarly demonstrated a relatively higher prevalence of missing and malformed lateral incisors in orthodontic populations [6]. Such anomalies create difficulties in space management, smile esthetics, and occlusal finishing, thereby increasing the need for reliable tooth-size prediction methods.

Management of missing or malformed anterior teeth often requires an interdisciplinary approach involving orthodontics and restorative dentistry. Treatment options generally include orthodontic space closure with canine substitution or orthodontic space opening followed by prosthetic replacement [5,7]. Accurate prediction of mesiodistal tooth width is critical in both approaches to achieve proper esthetic proportions, arch coordination, and functional occlusion. Incorrect predictions may lead to residual spacing, black triangles, midline discrepancies, and compromised esthetic outcomes.

Several methods have been proposed for evaluating tooth-size relationships and predicting missing tooth dimensions. Bolton’s analysis remains the gold standard for assessing interarch tooth-size discrepancy [1]. Regression-based methods such as Tonn’s formula and Abhi’s formula were later introduced to estimate incisor widths using measurements from the opposing arch [8,9]. Although these methods are relatively simple and clinically convenient, their applicability across different ethnic and regional populations remains uncertain. A previous study demonstrated significant ethnic variation in tooth-size relationships and questioned the universal applicability of Bolton’s standards [10]. Similarly, significant prediction errors were reported when Tonn’s and Abhi’s formulas were applied to an Iraqi population [11]. Indian studies have also demonstrated regional variations in Bolton’s ratios and tooth-size relationships [12].

Despite the widespread use of Bolton’s analysis and regression-based prediction methods in orthodontic practice, limited evidence is available regarding their applicability in the South Andhra population. Considering the known ethnic and regional variations in tooth dimensions, validation of these methods in specific populations becomes essential before routine clinical application. Therefore, the present study was undertaken with the following objectives: (1) to evaluate the applicability of Bolton’s anterior ratio in the present cohort, (2) to assess the accuracy of Tonn’s and Abhi’s formulas, and (3) to derive population-specific regression equations where necessary.

## Materials and methods

Study design

**Figure 1:**
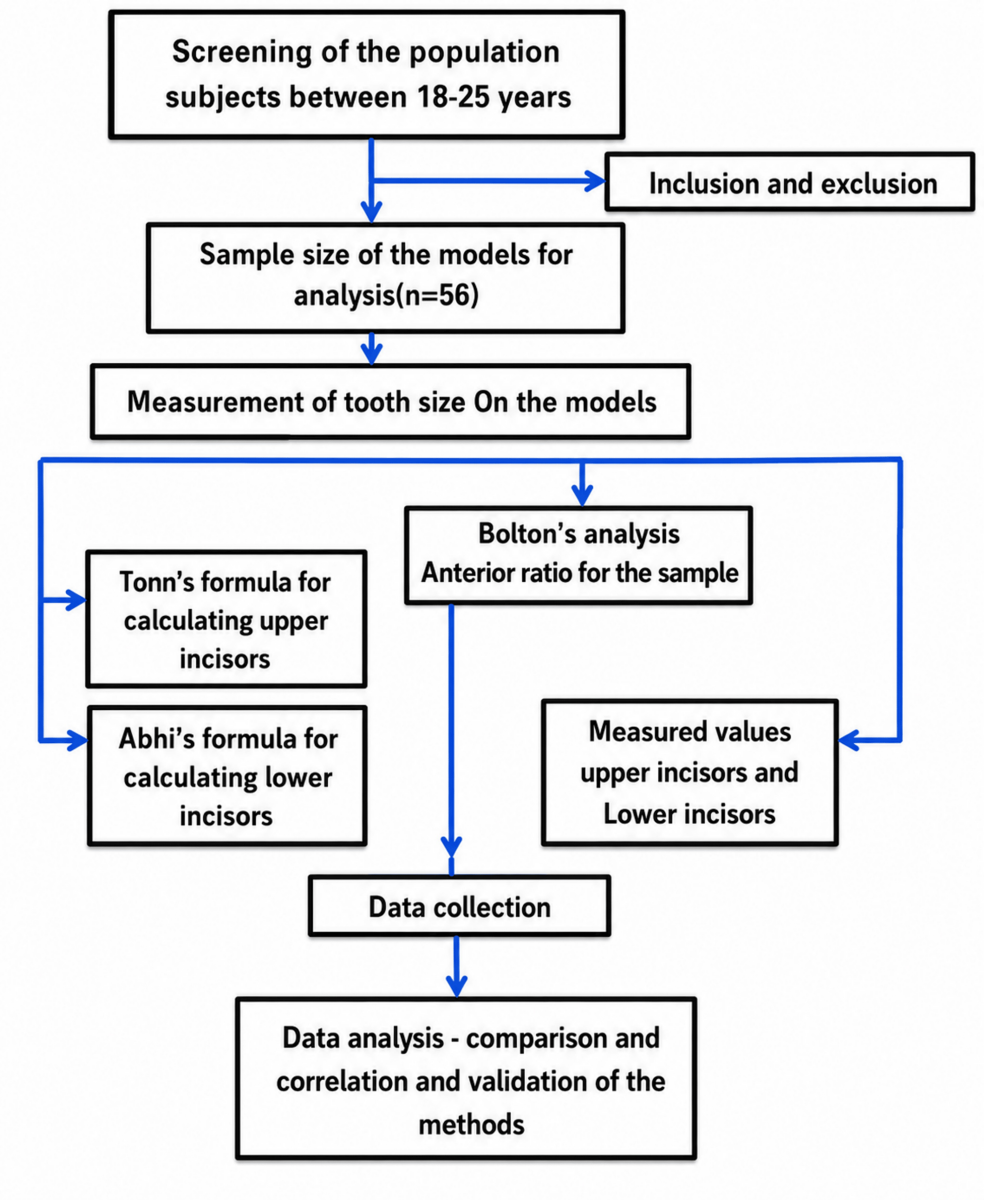
Flowchart of the study design, participant selection, measurement protocol, and statistical analysis

The present study was designed as an observational validation study conducted on maxillary and mandibular dental study models obtained from young adult subjects. The study aimed to evaluate the applicability of Bolton’s anterior ratio and the accuracy of regression-based tooth-size prediction methods in the study population. The study design, participant selection, measurement protocol, and statistical workflow are summarized in Figure 1.

Ethical approval

Ethical approval for this study was obtained from the Institutional Ethics Committee of Narayana Dental College and Hospital, Nellore, Andhra Pradesh, India (IEC/NDCH/2025/MAY-JULY/P-81, Version 01). The study protocol was considered exempt from full ethical review, as it involved measurements performed on dental study models without any intervention or risk to participants. Written informed consent was obtained from all participants prior to inclusion in the study.

Sample size estimation

Sample size estimation was performed based on validation of tooth-size prediction methods by comparing predicted and actual mesiodistal tooth widths using a paired t-test. The minimum detectable mean difference was adopted from the previous study [11], while the pooled standard deviation was derived from previously published literature [2]. Assuming a significance level of 5% and statistical power of 80%, the minimum required sample size was calculated as 53 subjects. A total of 56 eligible subjects were ultimately included in the study. Although the minimum required sample size was 53 subjects, 56 eligible subjects meeting the selection criteria were available and were included to maximize the available data and improve the precision of the estimates.

Study population

The study sample was obtained from young adults aged 18-25 years from Andhra Pradesh, India. Subjects were selected based on predefined inclusion and exclusion criteria. Only subjects demonstrating Angle’s Class I occlusion with minimal crowding or spacing (<3 mm) and clinically acceptable occlusal relationships were included to minimize the influence of severe malocclusion on tooth-size relationships.

Inclusion and exclusion criteria

Subjects aged between 18 and 25 years with fully erupted permanent dentition excluding third molars were included in the study. Individuals exhibiting Angle’s Class I occlusion with minimal crowding or spacing, normal overjet and overbite, and well-aligned dental arches were selected. Subjects with previous orthodontic treatment, missing or impacted anterior teeth, supernumerary teeth, extensive restorations, proximal attrition, craniofacial anomalies, periodontal disease, or distorted dental casts were excluded from the study.

Study model preparation and tooth measurements

**Figure 2:**
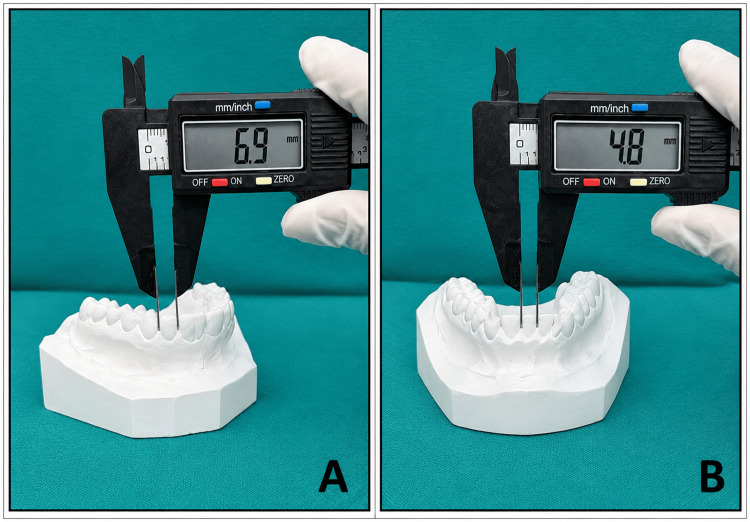
Measurement of mesiodistal tooth widths on maxillary and mandibular dental study models using a digital Vernier caliper (A) Maxillary cast; (B) mandibular cast. Measurements were recorded at the greatest mesiodistal dimension between the mesial and distal contact points of each tooth crown.

Maxillary and mandibular impressions were obtained using alginate impression material and poured using dental stone to prepare study models. Mesiodistal tooth dimensions were measured manually using an electronic digital Vernier caliper with an accuracy of 0.01 mm. Measurements were recorded at the maximum mesiodistal dimension between the mesial and distal contact points of the crown, as shown in Figure 2.

Mesiodistal widths of the maxillary and mandibular anterior teeth were measured bilaterally. The summed mesiodistal widths of the anterior teeth in both arches were calculated for further analysis.

Tooth-size analysis and prediction methods

Mesiodistal tooth widths were measured using a digital Vernier caliper. Bolton’s anterior ratio was calculated to evaluate the proportional relationship between the maxillary and mandibular anterior teeth using the following formula:

$$\text{Anterior Ratio} = \left( \frac{\text{Sum of mandibular anterior tooth widths}}{\text{Sum of maxillary anterior tooth widths}} \right) \times 100$$

The obtained values were compared with Bolton’s standard anterior ratio of 77.2% [1].

Tonn’s formula was used to predict the combined mesiodistal width of the maxillary incisors from mandibular incisor measurements [8]:

$$\text{Predicted Maxillary Incisor Width} = \left( \frac{4}{3} \times \text{Mandibular Incisor Width} \right) + 0.5$$

Abhi’s formula was used to predict mandibular incisor width from maxillary incisor measurements [9]:

$$\text{Predicted Mandibular Incisor Width} = \left( \text{Maxillary Incisor Width} - 0.5 \right) \times 0.75$$

The predicted values obtained using Tonn’s and Abhi’s formulas were compared with the corresponding actual measurements to evaluate their accuracy and clinical applicability in the study population. In addition, regression analysis was performed to derive prediction equations for tooth-size estimation in the study population. The resulting regression equations were evaluated by comparing the predicted values with the actual measurements and assessing the strength of association between the variables.

Reliability assessment

All measurements were initially performed by the primary investigator (BB). Examiner calibration was established with a senior researcher (SGS) before the final measurements. To assess measurement reliability, 10 randomly selected study models were remeasured after a one-week interval. Intraexaminer reliability was assessed from repeated measurements by the primary investigator, while interexaminer reliability was assessed by comparison with measurements made by the calibrated senior researcher. Reliability was evaluated using the intraclass correlation coefficient (ICC). The intraexaminer ICC was 0.98, and the interexaminer ICC was 0.96, indicating excellent agreement. Since excellent reliability was established, the measurements recorded by the primary investigator were used for the final statistical analysis.

Statistical analysis

Data were entered into Microsoft Excel (Microsoft Corp., Redmond, WA, USA) and analyzed using DATAtab statistical software (DATAtab e.U., Graz, Austria). Normality of the data was assessed using the Shapiro-Wilk test. Descriptive statistics, including mean, standard deviation, and range, were calculated for all measured and predicted parameters.

Paired t-tests were used to compare actual and predicted tooth dimensions and to evaluate differences between measured anterior ratios and Bolton’s standard values. Pearson correlation analysis was performed to assess the relationship between actual and predicted measurements. Bland-Altman analysis was used to evaluate agreement and prediction bias between methods. Linear regression analysis was performed to derive population-specific regression equations. A p-value of less than 0.05 was considered statistically significant.

## Results

A total of 56 study models were included in the study. The sample consisted of models of 36 women (64.28%) and 20 men (35.72%), with a mean age of 22.75 ± 2.55 years.

The descriptive statistics of individual maxillary and mandibular anterior tooth widths are presented in Table 1. Among the maxillary teeth, the central incisors demonstrated the greatest mesiodistal widths, whereas the mandibular central incisors exhibited the smallest dimensions. Bilateral symmetry was observed between the right and left corresponding teeth, with only minimal variation in mean tooth widths.

**Table 1:** Descriptive statistics of measured individual tooth widths SD: standard deviation; Max: maxillary; Man: mandibular; RCI: right central incisor; LCI: left central incisor; RLI: right lateral incisor; LLI: left lateral incisor; RC: right canine; LC: left canine

Parameter	Symbol	Mean ± SD	Minimum	Maximum
Maxillary right central incisor	Max (RCI)	8.44 ± 0.69 mm	7.4 mm	10.0 mm
Maxillary left central incisor	Max (LCI)	8.47 ± 0.70 mm	7.4 mm	10.0 mm
Maxillary right lateral incisor	Max (RLI)	7.46 ± 0.61 mm	5.8 mm	9.8 mm
Maxillary left lateral incisor	Max (LLI)	7.47 ± 0.61 mm	5.8 mm	9.8 mm
Maxillary right canine	Max (RC)	7.67 ± 0.53 mm	6.5 mm	8.9 mm
Maxillary left canine	Max (LC)	7.60 ± 0.56 mm	6.5 mm	8.9 mm
Mandibular right central incisor	Man (RCI)	5.53 ± 0.35 mm	4.8 mm	6.2 mm
Mandibular left central incisor	Man (LCI)	5.55 ± 0.38 mm	4.8 mm	6.2 mm
Mandibular right lateral incisor	Man (RLI)	6.86 ± 0.45 mm	6.1 mm	7.8 mm
Mandibular left lateral incisor	Man (LLI)	6.85 ± 0.44 mm	6.1 mm	7.8 mm
Mandibular right canine	Man (RC)	6.57 ± 0.57 mm	5.7 mm	7.7 mm
Mandibular left canine	Man (LC)	6.60 ± 0.58 mm	5.7 mm	7.7 mm

The descriptive statistics of the summed, calculated, and predicted parameters are shown in Table 2. The mean sum of maxillary anterior tooth widths was 44.75 ± 2.05 mm, whereas the mean sum of mandibular anterior tooth widths was 35.05 ± 1.87 mm. The mean measured maxillary incisor sum was 31.84 ± 1.62 mm, while the mean measured mandibular incisor sum was 24.79 ± 1.48 mm. The mean actual anterior Bolton’s ratio observed in the present cohort was 78.02% ± 2.05%, which was higher than Bolton’s original standard value of 77.2%.

**Table 2:** Descriptive statistics of summed, calculated, and predicted parameters SD: standard deviation; AR: anterior Bolton’s ratio; X: measured maxillary incisor width; Y: measured mandibular incisor width; X′: predicted maxillary incisor width; Y′: predicted mandibular incisor width

Parameter	Symbol	Mean ± SD	Minimum	Maximum
Sum of total maxillary anterior teeth	Σ Max Ant	44.75 ± 2.05 mm	41.8 mm	51.0 mm
Sum of total mandibular anterior teeth	Σ Mand Ant	35.05 ± 1.87 mm	32.0 mm	40.5 mm
Sum of measured maxillary incisors	Σ Max (X)	31.84 ± 1.62 mm	27.7 mm	36.9 mm
Sum of measured mandibular incisors	Σ Mand (Y)	24.79 ± 1.48 mm	21.6 mm	28.5 mm
Actual anterior Bolton’s ratio	Actual AR	78.02% ± 2.05%	73.20%	85.30%
Predicted maxillary incisor width using Tonn’s formula	Tonn (X′)	33.51 ± 1.97 mm	29.2 mm	39.5 mm
Predicted mandibular incisor width using Abhi’s formula	Abhi (Y′)	24.12 ± 1.25 mm	20.4 mm	27.3 mm

Comparison between actual and predicted values demonstrated statistically significant differences for Bolton’s anterior ratio and both regression-based prediction methods (Table 3). The actual anterior Bolton’s ratio was significantly higher than Bolton’s standard value, with a mean difference of 0.82 ± 2.05 and 95% confidence interval (CI) of 0.29 to 1.35 (p = 0.003). Tonn’s formula significantly overestimated maxillary incisor width by 1.67 ± 1.24 mm with 95% CI of 1.34 to 2.00 mm (p < 0.001), whereas Abhi’s formula significantly underestimated mandibular incisor width by −0.67 ± 0.98 mm with 95% CI of −0.93 to −0.41 mm (p < 0.001).

**Table 3:** Comparison between actual measurements, predicted values, and Bolton’s standard Paired t-test was used for comparison analysis. SD: standard deviation; CI: confidence interval *p-values less than 0.05 are statistically significant. Positive values indicate overestimation, whereas negative values indicate underestimation.

Comparison	Mean difference ± SD	95% CI	t-value	p-value
Actual anterior Bolton’s ratio vs. Bolton’s standard	0.82 ± 2.05	0.29 to 1.35	3.12	0.003*
Actual vs. Tonn’s predicted maxillary width	1.67 ± 1.24	1.34 to 2.00	8.41	<0.001*
Actual vs. Abhi’s predicted mandibular width	-0.67 ± 0.98	-0.93 to -0.41	-5.14	<0.001*

**Figure 3:**
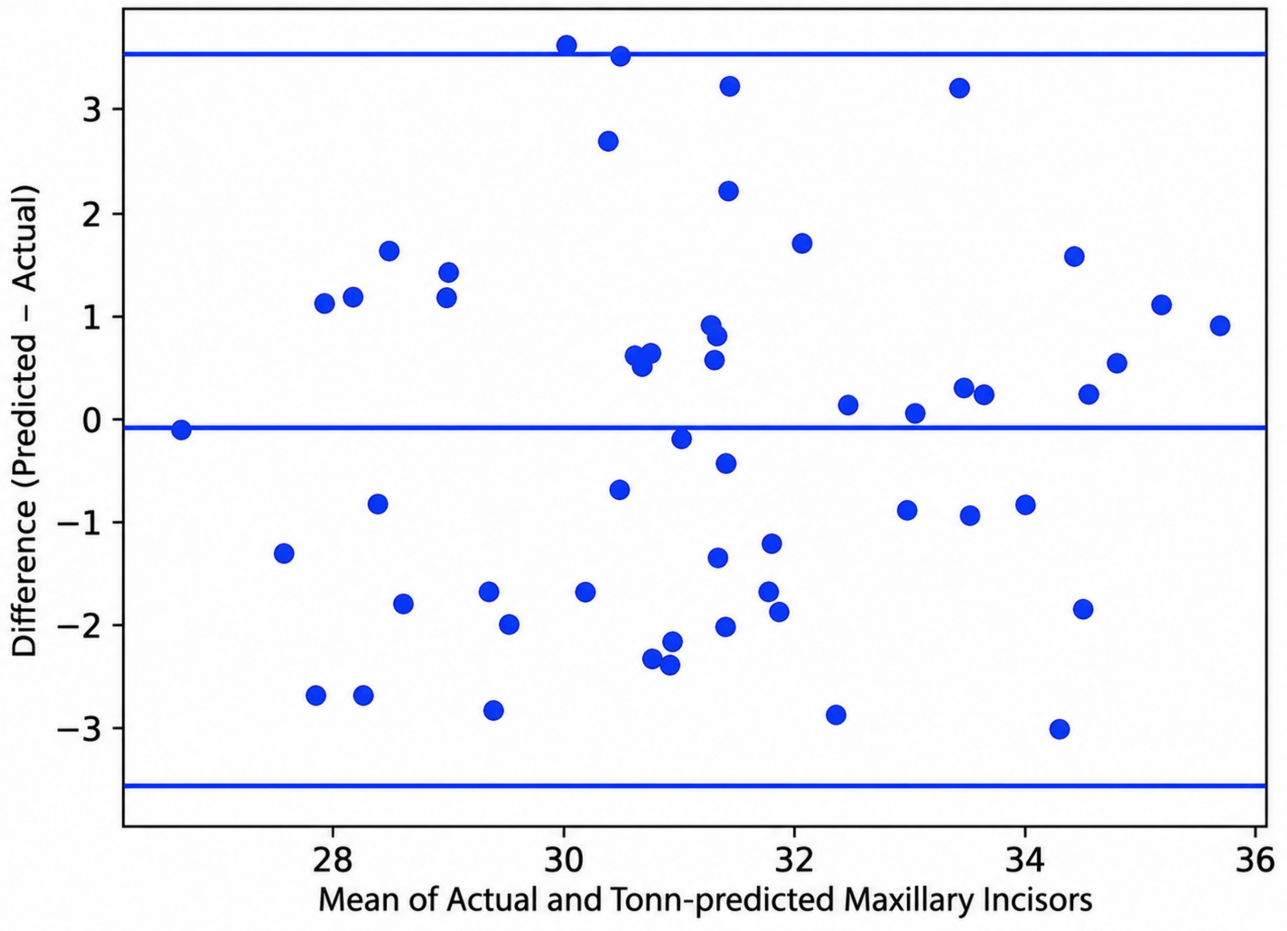
Bland-Altman plot showing agreement between actual and predicted maxillary incisor widths using Tonn’s formula The central solid line represents the mean bias (+1.67 mm), while the upper and lower dashed lines represent the 95% limits of agreement (−0.30 mm to +3.70 mm).

**Figure 4:**
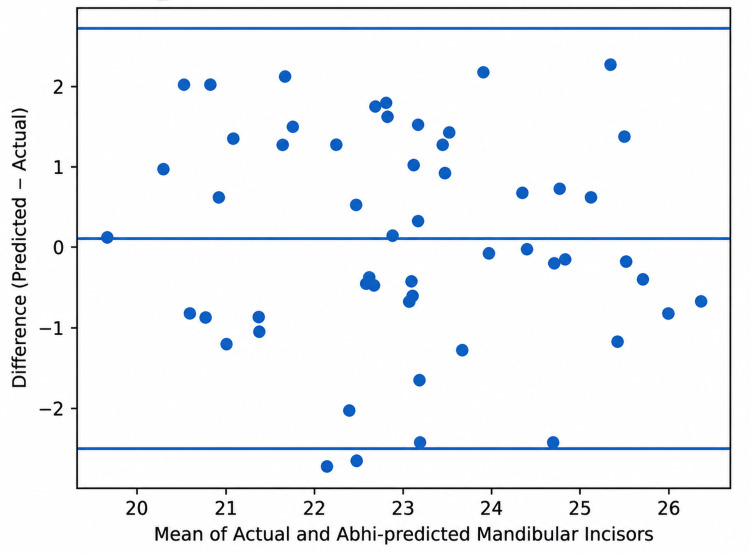
Bland-Altman plot showing agreement between actual and predicted mandibular incisor widths using Abhi’s formula The central solid line represents the mean bias (−0.67 mm), while the upper and lower dashed lines represent the 95% limits of agreement (−2.40 mm to +1.10 mm).

Correlation analysis demonstrated strong positive correlations between actual and predicted tooth widths for Tonn’s formula (r = 0.92; p < 0.001) and Abhi’s formula (r = 0.90; p < 0.001). Bland-Altman analysis showed a mean bias of +1.67 mm for Tonn’s formula with 95% limits of agreement from −0.30 mm to +3.70 mm, and a mean bias of −0.67 mm for Abhi’s formula with 95% limits of agreement from −2.40 mm to +1.10 mm (Table 4; Figures 3, 4).

**Table 4:** Correlation and Bland-Altman agreement analysis of prediction methods r: Pearson correlation coefficient; mm: millimeters *p-values less than 0.05 are statistically significant. Positive bias indicates overestimation, whereas negative bias indicates underestimation.

Method	Predicted segment	Pearson r	p-value	Mean bias	Lower limit of agreement	Upper limit of agreement	Interpretation
Tonn’s formula	Maxillary incisors	0.92	<0.001*	+1.67 mm	-0.30 mm	+3.70 mm	Overestimation
Abhi’s formula	Mandibular incisors	0.90	<0.001*	-0.67 mm	-2.40 mm	+1.10 mm	Underestimation

Linear regression analysis demonstrated a significant relationship between the sum of maxillary incisor widths and the sum of mandibular incisor widths (r = 0.725, R² = 0.526, standard error of estimate (SEE) = 0.92 mm) (Table 5).

**Table 5:** Linear regression equations for the prediction of the sum of maxillary and mandibular incisor widths X: measured sum of maxillary incisor widths; Y: measured sum of mandibular incisor widths; X′: predicted sum of maxillary incisor widths; Y′: predicted sum of mandibular incisor widths; r: Pearson correlation coefficient; R²: coefficient of determination; SEE: standard error of estimate; mm: millimeters

Prediction model	Regression equation	r	R²	SEE
Sum of mandibular incisor prediction	Y′ = 0.78X − 0.05	0.725	0.526	0.92 mm
Sum of maxillary incisor prediction	X′ = 0.95Y + 8.1	0.725	0.526	0.92 mm

## Discussion

Accurate evaluation of interarch tooth-size relationships is essential for orthodontic diagnosis, space analysis, finishing, and restorative treatment planning. Bolton’s analysis remains one of the most widely used methods for assessing tooth-size discrepancy; however, its universal applicability has been questioned because mesiodistal tooth dimensions vary among different ethnic and regional populations [1,10]. The present study evaluated the applicability of Bolton’s anterior ratio, Tonn’s formula, and Abhi’s formula in a young adult cohort and assessed whether population-specific regression equations could improve predictive accuracy.

The mean anterior Bolton’s ratio observed in the present cohort was 78.02% ± 2.05%, which was significantly higher than Bolton’s original standard value of 77.2% (p = 0.003). This finding suggests a relatively greater mandibular anterior tooth mass in relation to the maxillary anterior teeth when compared with Bolton’s original sample [1]. Simplified Bolton’s analysis has also shown clinical usefulness in Indian malocclusion groups, although regional validation remains important [2]. Previous studies demonstrated significant ethnic variation in interarch tooth-size relationships and questioned the universal applicability of Bolton’s standards [10]. Regional variation in Bolton’s ratios has also been reported in an Indian population, supporting the need for population-specific validation [12].

Tonn’s formula significantly overestimated maxillary incisor width by 1.67 mm (p < 0.001). Although the correlation between actual and predicted maxillary incisor widths was strong (r = 0.92), Bland-Altman analysis demonstrated a mean bias of +1.67 mm with wide limits of agreement from −0.30 mm to +3.70 mm. These findings indicate that Tonn’s formula follows the general trend of tooth-size variation but does not provide sufficient precision for individual clinical prediction. Similar prediction discrepancies have been reported when Tonn’s formula was applied to an Iraqi population [11].

Abhi’s formula significantly underestimated mandibular incisor width by 0.67 mm (p < 0.001). Although a strong positive correlation was observed between actual and predicted mandibular incisor widths (r = 0.90), Bland-Altman analysis demonstrated a mean bias of −0.67 mm with wide limits of agreement from −2.40 mm to +1.10 mm. Abhi’s formula was proposed as an Indian modification of Tonn’s approach [9]. However, the present findings indicate that even within Indian populations, regional variation may influence the accuracy of regression-based prediction methods. Similar Indian models, including the ViVan formula and the Schedule Variance formula, also emphasize the need for localized prediction methods [13,14]. The importance of indigenous prediction methods is further supported by the ViVan formula and the Schedule Variance formula proposed in Indian samples. The ViVan formula estimates maxillary lateral incisor width using the mandibular lateral incisor width with a population-specific correction factor [13]. Similarly, the Schedule Variance formula was proposed for estimating mandibular central incisor width in Indian subjects [14]. These localized approaches support the present finding that population-specific equations may be more clinically meaningful than universal prediction formulas.

An important observation in the present study was the difference between correlation and agreement. Both Tonn’s and Abhi’s formulas demonstrated strong positive correlations with actual tooth widths; however, Bland-Altman analysis revealed systematic bias and wide limits of agreement. This distinction is clinically important because correlation evaluates association, whereas agreement analysis evaluates whether two methods can be used interchangeably. It has been emphasized that two methods may correlate strongly despite showing poor clinical agreement [15].

Linear regression analysis demonstrated a moderate positive predictive relationship between the sum of maxillary and mandibular incisor dimensions (r = 0.725, R² = 0.526, SEE = 0.92 mm). The derived equations were as follows:

$$Y^\prime = 0.78X - 0.05$$

$$X^\prime = 0.95Y + 8.1$$

where Y' represents the predicted total width of mandibular incisors, X' represents the predicted sum of maxillary incisor widths, X represents the measured sum of maxillary incisor widths, and Y represents the measured total width of mandibular incisors.

Validation

The newly derived regression equation was further validated by calculating the predicted anterior Bolton’s ratio. The predicted anterior ratio was 78.11%, which showed no statistically significant difference when compared with the actual anterior Bolton’s ratio of 78.02% (p = 0.977), indicating good agreement between the predicted and actual values within the present sample. The SEE of the derived mandibular prediction equation was 0.92 mm, indicating acceptable predictive precision. The correlation coefficient was r = 0.73, with a coefficient of determination of R² = 0.53, indicating that approximately 53% of the variation in the sum of mandibular incisor widths could be explained by the sum of maxillary incisor widths.

These findings support the clinical usefulness of the population-specific regression equation in the present study population. Similar Indian models, such as the ViVan formula and the Schedule Variance formula proposed by Jadhav et al., also emphasize the need for localized prediction methods [13,14]. Population-based studies from Turkish, Japanese, and Peruvian samples have similarly demonstrated deviations from Bolton’s standards, further supporting population-specific interpretation of tooth-size relationships [16-18].

The clinical implications of the present findings are important in orthodontic diagnosis and interdisciplinary treatment planning. Since Bolton’s standard ratio, Tonn’s formula, and Abhi’s formula showed significant bias in the present cohort, these methods should be applied cautiously during anterior space assessment. Direct use of these formulas may lead to incorrect space allocation, residual spacing, black triangles, midline discrepancies, or compromised esthetic outcomes. The derived population-specific regression model may provide better guidance for anterior tooth-size prediction, especially in cases involving missing, peg-shaped, or malformed incisors requiring orthodontic space management or restorative planning.

The present study has certain limitations. Although the sample size was statistically adequate, the study was limited to a single-center young adult cohort with Angle’s Class I occlusion and minimal crowding or spacing. Therefore, the findings may not be directly generalizable to other malocclusion groups, mixed dentition patients, older age groups, or wider populations. Measurements were performed manually on dental study models, although high intraexaminer and interexaminer reliability was obtained. In addition, the derived regression equations were validated only within the present sample and require external validation before broader clinical application.

Future studies should include larger multicentric samples representing different geographic and ethnic populations. Evaluation of these prediction methods in various malocclusion groups and mixed dentition patients may provide broader clinical applicability. Further research using digital model analysis and artificial intelligence-based predictive approaches may improve the precision of tooth-size prediction methods. External validation of the derived regression equations is also necessary before routine clinical implementation.

## Conclusions

Within the limitations of the present study, Bolton’s anterior ratio, Tonn’s formula, and Abhi’s formula demonstrated significant prediction bias in the evaluated young adult cohort. The actual anterior Bolton’s ratio was significantly higher than Bolton’s standard value, while Tonn’s formula overestimated maxillary incisor widths and Abhi’s formula underestimated mandibular incisor widths. Although strong positive correlations were observed, Bland-Altman analysis revealed wide limits of agreement, indicating limited precision for individual clinical application. The derived population-specific regression equations demonstrated improved predictive agreement and may serve as a more reliable adjunct for orthodontic diagnosis, space analysis, and restorative treatment planning in the studied population.
